# Supplementary material for: Real-time assembly of ribonucleoprotein complexes on nascent RNA transcripts
Source: Nat Commun. 2018 Nov 30;9:5087. doi: 10.1038/s41467-018-07423-3 (PMC6269517; doi:10.1038/s41467-018-07423-3)
Supplement: Supplementary file 1 — Supplementary Information [file 41467_2018_7423_MOESM1_ESM.pdf]

Supplementary Information for

Real-time assembly of ribonucleoprotein complexes on nascent RNA transcripts

by Duss *et al*.

Supplementary Notes

Supplementary Note 1: Transcription complexes are structurally homogeneous and experience a similar fluorescence decay constant within the ZMW volume

A tightly distributed value for the normalized intensity change during transcription (= almost constant *ΔI(TK)/ΔI(diss)* for all the molecules of a specific length; Fig. 2g) means that the two Cy3 dyes located at the 3'-end of the DNA template travel a very similar absolute distance along the evanescent field during transcription for all the RNA molecules of a specific length (see mathematical derivation in Supplementary Fig. 2e). This implies that there is a structurally homogeneous population of transcription complexes in the different ZMWs, on the time scale of the acquisition dwell time of 100 ms, and thus, monitoring transcription by following the intensity change is a quantitative measure. In addition, it indicates that the transcription complexes are all located within an evanescent field with a similar decay constant normal to the surface (see mathematical derivation in Supplementary Fig. 2e). By modeling the excitation field as well as the efficiency of emission using a finite difference time domain approximation to Maxwell's electromagnetic equations (https://kb.lumerical.com/en/sp_fluorescence_enhancement.html), we can calculate the expected fluorescence as a function of the position within the ZMW (Supplementary Fig. 2b). From that we can calculate the position-dependent decay constant within the ZMW. We find that the decay constant is approximately constant within most of the ZMW volume (within 2-fold) except close to the SiO_2/Aluminum interface (*z*-position = 0 nm), as well as just outside the metal ZMW (SiO2/water interface) (Supplementary Fig. 2c,d). A transcription complex, which is immobilized away from the sidewall of the ZMW, is therefore expected to experience a similar decay constant irrespective of its axial positioning and its DNA length. Finally, the evanescent field decay constant is largely independent of the fluorophore color (Cy3 or Cy3.5) used to label the DNA template (compare Supplementary Figures 2c,d), in agreement with a normalized fluorescence intensity change during transcription that is very similar for Cy3- or Cy3.5 labeled DNA templates of a specific length (Fig. 2h).

Supplementary Note 2: Semi-empirical model for relating normalized intensity change during transcription with transcribed RNA length (explanations how to fit Fig. 2h)

To model the position change of the fluorescence dyes at the 3'-end of the DNA template as they move through the evanescent field during transcription, we used a worm-like chain (WLC) model including DNA flexibility [1]. According to this model, the root-mean-square end-to-end distance <*R(l)*> of the two dyes before and after transcription (which corresponds to the distance travelled along the evanescent field; *z1-z2* in Supplementary Fig. 2e) can be described by:

$$\langle R(l)\rangle = \sqrt{2Pl\left[1-\frac{P}{l}\left(1-e^{l/P}\right)\right]} \quad (1)$$

P is the DNA persistence length (50 nm = 150 bp) and l the DNA contour length (maximum physically possible extension), which corresponds to the distance travelled by the RNA polymerase along the DNA template during transcription. The expected normalized intensity change during transcription *ΔI (TK)/ΔI (diss)* along the evanescent field can then be described by:

$$\Delta I(TK)/\Delta I(diss) = 1 - e^{-c\langle R(l)\rangle} \quad (2)$$

See mathematical derivation in Supplementary Fig. 2e for details. c is the evanescent field decay constant and $\langle R(l)\rangle = z1\text{-}z2$. However, this model did not agree well with our data (Supplementary Fig. 2h).

During transcription, the two dyes at the 3'-end of the DNA template move closer to the surface but at the same time, the nascent RNA is elongating and, in principle, pushing the two dyes away from the surface. Yet, RNA shows an overall compaction in the millisecond time scale upon folding, thereby adopting a global shape close to the native one within less than a second [2]. Assuming a nascent RNA extension during transcription of 50 Å (15 bp in dsDNA dimensions) and being approximately independent of the transcribed RNA length [3], the position of the RNA polymerase may not substantially change during transcription (see Supplementary Fig. 2e as a cartoon representation).

The labeled DNA template is not directly attached to the ZMW surface, but is attached indirectly through the stalled or elongating transcription complex (macromolecular complex consisting of DNA template, RNA polymerase and nascent RNA), which is further hybridized by its nascent RNA 5'-end to a long tether. Apart from the hybridization region, this tether consists of a 154 bp double-stranded DNA with a biotin at the other end. This entire complex is then immobilized to the surface of the ZMW. Modeling the time-averaged (100 ms experimental dwell time) dimensions of this entire macromolecular assembly requires not only knowledge about the persistence length of the DNA transcription template and the long dsDNA tether, but also about the relative motions and structures of the other components in the system. In particular, the transcribing nascent RNA, which is folding co-transcriptionally, is difficult to model reliably.

We therefore modified equation (2) by introducing an additional empirical parameter $\langle d\rangle$, which is independent of the length of the transcribed RNA and unifies all the data which cannot be described by the transcribed-RNA-length dependent parameter $\langle R(l)\rangle$ into a single empirical model with $z1\text{-}z2 = \langle R(l)\rangle - \langle d\rangle$. We obtain:

$$\Delta I(TK)/\Delta I(diss) = 1 - e^{-c(\langle R(l)\rangle - \langle d\rangle)} \quad (3)$$

Within our experimental range of ~ 150-550 transcribed RNA nucleotides, the experimental data can be well described by fitting it to this modified equation (Supplementary Fig. 2i), and we obtain a decay constant c, which is in agreement with calculations of the decay constant within the ZMW holes using a finite difference time domain approximation to Maxwell's electromagnetic equations (Supplementary Figs. 2c,d and Supplementary Note 1). We note that this semi-empirical model does not appropriately describe the transcription of smaller RNAs extrapolated to zero length, partly because the assumption that RNA extension during transcription is independent of

the transcribed RNA length, which is represented by the constant <*d*>, does not hold anymore for small RNA molecules.

We find that a simpler empirical exponential model, which does not describe the DNA as a WLC but describes it as an extended rod, fits our data similarly well and also better describes the transcription of smaller RNAs extrapolated to zero length (Supplementary Fig. 2j). However, this simplified model yields a fluorescence decay constant, which is less in agreement with our calculations of the decay constant within the ZMW holes using a finite difference time domain approximation to Maxwell's electromagnetic equations.

Overall, a semi-empirical model, which treats the DNA as a WLC and includes an additional transcribed-RNA-length-independent parameter, describes our data well within our experimental range of ~ 150-550 transcribed RNA nucleotides and also correctly predicts the decay constant within the ZMW (Fig. 2h). Alternatively, the data can also be well-described by a simpler empirical model using a single exponential function with a single parameter (Supplementary Fig. 2j).

Supplementary Note 3: Slower Cy3 oligo hybridization rates compared to *Zhang et al.*

To verify transcription of full-length RNA, we used a Cy3 DNA oligo that can hybridize to the 3'-end of the nascent RNA. We used a DNA transcription template that was labeled with two Cy3.5 dyes at its 3'-end. In > 75 % of the single-molecule traces with transcription-dependent Cy3.5 intensity changes, a Cy3 signal appears shortly after template dissociation, in agreement with the bulk transcription experiments (Supplementary Fig. 1a,b). The rate of Cy3 oligo binding to the nascent RNA can be determined by fitting an exponential function to the distribution of times between end of transcription marked by the end of the Cy3.5 fluorescence increase, and the binding of the Cy3 oligo probe, corresponding to a bimolecular association rate constant of $5.2 \pm 0.1 \times 10^5\ M^{-1}\ s^{-1}$ (Supplementary Fig. 1f).
Notably, the Cy3 oligo binding on-rate to the nascent RNA is 10-fold slower compared to a previous study using single-molecule TIRF microscopy to measure the hybridization of the same Cy3 DNA oligo sequence to a short pre-transcribed RNA devoid of guanosine nucleotides to minimize secondary structure [4]. We do not see significantly different hybridization rates for the Cy3 oligo in TIRF compared to ZMW experiments. The slower on-rate we obtained may be due to different buffer compositions or the secondary structural context surrounding our Cy3 oligo binding site, which is in agreement with Zhang *et al*. also finding 10-fold slower on-rates by introducing a single guanosine into their short Cy3 oligo binding site [4].

Supplementary Note 4: Fitting transcription progression and stalling times

The time of transcription progression can reliably be separated from stalling of RNAP at both the 5'-end and 3'-end of the DNA template using appropriate labeling scheme, at faster transcription rates (Fig. 2e, Fig. 4 and Supplementary Fig. 8). For example, to

detect short stalling of the polymerase at the 3'-end, we labeled the DNA template with 2xCy3 or 2xCy3.5 dyes at the 3'-end. However, alternative labeling schemes (e.g. labeling the 5'-end of a large DNA template; Fig. 2d) can significantly hamper detection of 3'-end stalling. This is because the intensity may drop close to the baseline before the end of transcription such that a sudden intensity drop characteristic of RNAP dissociation may be difficult to detect. Furthermore, the presence of a protein binding non-specifically to the surface can result in baseline distortions. Finally, transcription of small constructs at very low NTP concentrations can lack a detectable intensity change during very slow transcription progression. Those situations complicate the separate detection of RNAP progression and stalling when observing a single molecule.

Nevertheless, we are able to obtain an average transcription rate and stalling time by fitting, for an ensemble of molecules, the total time of transcription progression and stalling at the 3'-end to an appropriate mathematical model: If the transition from a state A to state B is limited by a single kinetically-significant step, the waiting time distribution follows a single exponential decay. The joint probability of two sequential processes is a convolution of each individual process [5]. For a large number of sequential processes with the same rate constant, the distribution approximates a Gaussian distribution. Assuming that each NTP incorporation cycle can be described by a rate-limiting step with a similar rate constant, the distribution of the total times to transcribe an RNA can be described by a Gaussian distribution, which fits our data well (Fig. 3a). If dissociation of the RNAP at the 3'-end can be approximated by a single rate-limiting step, the RNAP waiting times before dissociation can be described by a single exponential distribution. Transcription and stalling of the RNAP at the 3'-end are two sequential processes. Hence, the total time of transcription progression including stalling of the RNAP at the 3'-end can be approximated by a convolution of a Gaussian (to describe transcription) and an exponential (to describe subsequent RNAP dissociation during termination) [5]. Indeed, we found that by fitting the total time of transcription progression including stalling at the 3'-end to a convolution of a Gaussian and an exponential yields very similar results as when fitting transcription progression and stalling separately to a Gaussian and an exponential, respectively.

<u>Supplementary Note 5</u>: Monitoring transcription of RNAs of > 500 or < 100 nts in real-time

Due to the exponentially decaying excitation profile within the zero-mode waveguides, transcription of RNAs of 200-500 nucleotides can be detected with high sensitivity (Fig. 2h and Supplementary Fig. 8) using a DNA template labeled at the 3’-end. For monitoring transcription progression for RNAs larger than 500 nucleotides it is more effective to label the DNA template at both termini with different colors allowing monitoring of transcription of the first and last 500 nucleotides by observing the fluorophore at the 5'- or 3'-end, respectively. Long RNAs of several thousand nucleotides should also be accessible with our approach, however, monitoring transcription progression in real-time only of the first and last 500 nucleotides.

On the other end, RNAs significantly shorter than 100 nucleotides, will generate only a small intensity change during transcription progression, which may become difficult to detect. Also, the very short transcription time of small RNAs at fast transcription rates

may result in an underestimation of the transcription rate (Fig. 3b). Approximate transcription rates of such small RNAs could still be estimated by inferring the end of transcription by the dissociation of the DNA template using appropriate transcription terminators with fast dissociation rates (Fig. 4a). Such an estimate for the end of transcription is still more accurate compared to the usage of labeled DNA oligonucleotides hybridizing to the 3'-end of nascent RNAs, especially when biologically relevant RNAs with secondary structure are investigated. We found that the on-rate for the labeled DNA oligonucleotide hybridization becomes slower (average arrival time of 20 seconds using 100 nM Cy3 oligo, see Supplementary Fig. 1f and Supplementary Note 3) compared to artificially designed transcription templates used in previous studies [4,6]. Complementary to our approach, detection of transcription progression of very small RNAs (< 30 nucleotides) in real-time is possible by following a FRET efficiency change between a Cy3 labeled 3'-end of the DNA transcription template and a Cy5 labeled RNAP [7,8].

Supplementary Note 6: Modulating RNAP dissociating kinetics at 3'-end of transcription template

Using our standard construct containing a triple terminator (*M13central++*, *rrnBT1* and *T7Te*) followed by two Cy3 dyes (Supplementary Fig. 3a) at the 3' terminus, we found fast RNAP dissociation kinetics with RNAP residing at the 3' terminus for 3.2 ± 0.8 seconds on average after the end of transcription (Fig. 4c).

To modulate the RNAP stalling duration at the 3'-end of the DNA transcription template, these experiments were repeated with a construct containing a single *T7Te* transcription terminator directly followed by a single-strand break in the non-template strand and the two bulky Cy3 dyes attached on the opposite template strand (Supplementary Fig. 3b). In this latter construct, the RNAP is not expected to terminate efficiently because the Cy3 dyes are predicted to clash with the DNA entry channel, preventing the active site of the polymerase from reaching the transcription-terminating nucleotide (Supplementary Fig. 3b). While the fraction of molecules having very long RNAP stalling of several minutes (Fig. 4b) was similar to the triple terminator construct (5-15 %), the average residence of the fast dissociating RNA polymerase molecules at the blocked terminator was increased to 20 ± 5 seconds (Fig. 4c). These results demonstrate that the dissociation kinetics of the RNA polymerase are influenced by the structural context at the 3'-end of the DNA template.

Supplementary Note 7: Detecting Cy5-S15 photobleaching

Among all the traces containing at least one stable binding event (N-class RNA molecules), 25-50 % have the last S15 binding event limited by the end of the 10-minute movie, while another 30-60 % have the last S15 binding event limited by photobleaching. For the latter case, we can in principle not distinguish Cy5-S15 photobleaching from Cy5-S15 dissociation. For the traces that we assign as containing a Cy5-S15 photobleaching event, we do not find any Cy5-S15 rebinding events following disappearance of the Cy5-S15 bound signal till the end of the recording time. We define the time after Cy5-S15 disappearance (with a S15-bound lifetime of at least 10

seconds) until the end of the 10 minutes recording time to be at least 200 seconds in order to assign those events to photobleaching limited events. This period of no Cy5-S15 binding could either be due to a photobleached S15 protein bound to the RNA preventing rebinding of another Cy5-S15 protein, but could also be due to an RNA conformational rearrangement to an S15 binding incompetent state. To distinguish between these two cases, we repeated our experiment with a *delta-A747* RNA mutant, which has been shown to have a lower affinity for S15 [9,10]. Indeed, in contrast to the *wild-type* RNA, we obtain a shorter average Cy5-S15 bound lifetime (Supplementary Fig. 6a,b) with multiple rebinding events per trace (Supplementary Fig. 4), the fraction of molecules for which we do not see a rebinding event for at least 200 seconds after the last Cy5-S15 signal disappearance (what we defined above as being due to photobleaching), drops below 5% compared to 60 % for *wild-type* RNA at the same 25 nM Cy5-S15 concentration. We can exclude that this is due to more efficient RNA folding of the *delta-A747* single nucleotide deletion mutant compared to *wild-type* RNA, because the relative populations of the nascent RNA molecules binding S15 stably, transiently or not at all are unchanged (Supplementary Fig. 6g) suggesting that RNA folding efficiency is unaffected by the A747 deletion. Overall, this argues that 30-60 % of the class-N traces in the *wild-type* RNA have the last Cy5-S15 binding event limited by photobleaching.

<u>Supplementary Note 8</u>: Comparison of S15 binding and dissociation rate constants of N-class RNA molecules to previous literature

To calculate the average S15-bound lifetime, we fitted the distribution of lifetimes to an exponential function (Supplementary Fig. 6a,b). For the natively folded (N-class) RNA molecules, we find a S15 dissociation rate constant $k_{off} = 6.0 \pm 0.7 \times 10^{-3}\ s^{-1}$, or average S15-bound lifetime of around 170 seconds, which represents an upper limit for the dissociation rate because many binding events are limited by photobleaching or the end of the 10-minute movie (see Supplementary Note 7).

To estimate the average S15 binding rate constant, we fit the distribution of Cy5-S15 arrival times either from Cy5-S15 protein delivery to first stable Cy5-S15 binding event (for pre-transcribed and pre-folded full-length RNA) or the times from end of transcription till first stable Cy5-S15 binding event (for nascently transcribed RNA), respectively. For the pre-folded RNA experiment, we had to account for a short delay due to mixing upon protein delivery to the chip, by including an additional parameter in the fit (Supplementary Fig. 7a). For both the pre-folded RNA and nascently transcribed RNA experiments, the data was fit well by a double exponential function, revealing an additional small population with a slower on-rate. For the main phase, we obtained a $k_{on} = 5.4 \pm 0.3 \times 10^6\ M^{-1}\ s^{-1}$ (86 %) (Supplementary Fig. 7a) or $k_{on} = 6.0 \pm 0.4 \times 10^6\ M^{-1}\ s^{-1}$ (68 %) (Supplementary Fig. 7b) for Cy5-S15 binding to a pre-folded or to a nascently transcribed RNA, respectively.

The dissociation and association rates are in a similar range with slightly higher on-rates as measured previously with different ensemble techniques using *in vitro* pre-transcribed and pre-folded RNA and consisting of different minimal constructs of the central domain and S15 from *Bacillus stearothermophilus* (in contrast to the *E. coli* system used here): $k_{on} = 6 \times 10^5\ M^{-1}\ s^{-1}$ and $k_{off} = 5 \times 10^{-3}\ s^{-1}$; using a microfluidic reactor and no monovalent or divalent cations [11], $k_{on} = 4.4 \times 10^5\ M^{-1}\ s^{-1}$ and $k_{off} = 1 \times 10^{-4}\ s^{-1}$; using EMSA in

presence of 50 mM K-acetate and 10 mM Mg^{2+} [12] and $k_{off} = 1 \times 10^{-4}\ s^{-1}$; using single-molecule total internal reflection microscopy and fluorescent labeling of the pre-transcribed and pre-folded RNA but not direct observation of S15 binding [13]. Our slightly higher on-rates could either be explained by surface effects of the ZMWs, or by the different buffer conditions compared to previous experiments. Considering that the nascent or pre-transcribed RNAs are immobilized through a 150 nt long biotin-containing dsDNA linker (hybridized to the 5'-end of the nascent RNA), protein binding does not occur in close proximity to the negatively charged surface of the ZMW holes and therefore, the on-rates are likely not affected. In contrast, it has been shown that buffer composition significantly affects S15 binding on-rates. Our buffer consists of 50 mM Tris (pH 7.5), 14 mM $MgCl_2$, 20 mM NaCl, 0.04 mM EDTA, 40 µg/ml BSA, 0.01 % Triton X-100, 2 mM spermidine, 1 mM putrescine, 150 mM KCl, 2 mM DTT, 0.25 % each Biolipidure 203 and 206, 0.5 mg/ml tRNA yeast, 0.5 % Tween-20, 0.1 mg/ml casein #37582. Previous studies have different experimental conditions, explaining the variation in rate constants observed between different studies: The presence of K-acetate decreases the on-rate 30-fold [11], the absence of Mg^{2+} decreases the on-rate >200-fold [12] and the presence of Nonidet-40 increases the affinity almost 10-fold [14] indicating that the buffer conditions can significantly influence the rate constants. The new reported values are consistent with the ranges previously observed in a variety of contexts, and with the general expectations for bimolecular association rates and dissociation rates for RNA-protein complexes.

Supplementary Methods

Overview of constructs used in this study

Autosticky PCR to generate the single-stranded overhangs for labeling the DNA transcription templates was performed using following primers:

5'-autosticky PCR primer:
5'-TCACG AAAGC TGAGT AGTCA CGAGT CTTCT /idSp/ ctggcagttttagg ctgatttgg-3'

3'-autosticky PCR primer:
5'-CCttaatcatactcaccaaattaccatcCC /idSp/ ATAAACGCAGAAAGGCCCAC -3'

idSp denotes an abasic site.

Following DNA oligonucleotides were hybridized to the single-stranded overhangs of the DNA transcription templates for their labeling:

Labeling 5'-end of DNA template:
p17_Cy5: 5'- Cy5- AGA AGA CTC GTG ACT ACT CAG CTT TCG TGA -3'
p21_Cy3: 5'- Cy3- AGA AGA CTC GTG ACT ACT CAG CTT TCG TGA -3'
p24_Cy3/5: 5'- Cy3- AGA AGA CTC GTG ACT ACT CAG CTT TCG TGA -Cy5-3'

Labeling 3'-end of DNA template:
p71_Cy5: 5'-Cy5-GGG ATG GTA ATT TGG TGA GTA TGA TTA AGG -3'
p72_2xCy3: 5'-Cy3-GGG ATG G /iCy3/ TA ATT TGG TGA GTA TGA TTA AGG-3'
p88_2xCy3p5: 5'-Cy3.5-GGG ATG GTA ATT TGG T /iCy3.5/ GA GTA TGA TTA AGG -3'

Following sequences for the DNA transcription templates were ordered from IDT as geneBlocks (these are the templates used during autosticky PCR with the primers mentioned above):

All sequences have following common backbone:
RNAP promoter (green), hybridization sequence for immobilization of stalled complex through 5'-end of nascent RNA (red), potential binding site for DNA oligo binding to 5'-end of nascent RNA (blue), sequence of interest (see further below), binding site for labeled DNA oligo binding to 3'-end of nascent RNA (orange) and triple transcription terminator (black).

This is the DNA sequence:
ctggcagttt taggctgatt tggttgaatg ttgcgcggtc agaaaattat tttaaatttc ctcttgtcag gccggaataa ctccctataa tgcgccacc
ACT ACCAC CACCC AACCA ACACA CC AAC CAC TCC AAT TAC ATA CAC C

(Sequence of interest)
CCCTA TCCCT TATCT TAAC
GGCTCCTTTTGGAGCCTTTTTTTTTGGAGATTTTCTAAAACGAAAGGCTCAGT
CGAAAGACTGGGCCTTTCGTTTTATCT TAATC ACACT GGCTC ACCTT
CGGGT GGGCC TTTCT GCGTT TAT

The DNA transcription template sequence for the blocked 1x-terminator is identical except that the triple-terminator sequence is replaced by following sequence (T7Te terminator):
TAATC ACACT GGCTC ACCTT CGGGT GGGCC TTTCT GCGTT TAT

These are the inserted sequences of interest (the name corresponds to the number of transcribed nucleotides after addition of the NTPs to the stalled complex till transcription termination at the first terminator):

<u>517 nts construct</u>:
tgacgggggc ccgcacaagc ggtggagcat gtggtttaat tcgatgcaac gcgaagaacc
ttacctggtc ttgacatcca cggaagtttt cagagatgag aatgtgcctt cgggaaccgt
gagacaggtg ctgcatggct gtcgtcagct cgtgttgtga aatgttgggt taagtcccgc
aacgagcgca acccttatcc tttgttgcca gcggtccggc cgggaactca aaggagactg
ccagtgataa actggaggaa ggtggggatg acgtcaagtc atcatggccc ttacgaccag
ggctacacac gtgctacaat ggcgcataca aagagaagcg acctcgcgag agcaagcgga
cctcataaag tgcgtcgtag tccggattgg agtctgcaac tcgactccat gaagtcggaa
tcgctagtaa tcgtggatca gaatgccacg gtgaatacgt tcccgggcct tgtaca

<u>388 nts construct</u>:
ggcgtaaagc gcacgcaggc ggtttgttaa gtcagatgtg aaatccccgg gctcaacctg
ggaactgcat ctgatactgg caagcttgag tctcgtagag gggggtagaa ttccaggtgt
agcggtgaaa tgcgtagaga tctggaggaa taccggtggc gaaggcggcc ccctggacga
agactgacgc tcaggtgcga aagcgtgggg agcaaacagg attagatacc ctggtagtcc
acgccgtaaa cgatgtcgac ttggaggttg tgcccttgag gcgtggcttc cggagctaac
gcgttaagtc gaccgcctgg ggagtacggc cgcaaggtta aaactc

<u>247 nts construct</u>:
tgacgggggc ccgcacaagc ggtggagcat gtg TCAC cac gtgctacaat ggcgcataca aagagaagcg
acctcgcgag agcaagcgga cctcataaag tgcgtcgtag tccggattgg agtctgcaac tcgactccat
gaagtcggaa tcgctagtaa tcgtggatca gaatgccacg gtgaatacgt tcccgggcct tgtaca

<u>199 nts construct</u>:
tgacgggggc ccgcacaagc ggtggagcat gtg TCAC cac gtgctacaat ggcgca cTCACg
tgcgtcgtag tccggat tgg cTTCGg ccat gaagtcggaa tcgctagtaa tcgtggatca gaatgccacg
gtgaatacgt tcccgggcct tgtaca

<u>171 nts construct</u>:

GGCgcac gcaggcggtt tgCCGGAAACGGcaag cttgagtctc gtagaggggg gtagaattcc agGAAA ctg gaggaatacc ggtggcgaag gcggccccct ggacgaagac tgacgctcag gtgcGCC

The stalled complex was immobilized through the nascent RNA with the following double-stranded DNA with single-stranded overhang:

The single-stranded overhang was generated with autosticky PCR and following two primers:
5'-primer: 5'-BiotinTEG/CG GGC CTC TTC GCT ATT AC-3'
3'-primer: 5'-GGT GTG TTG GTT GGG TGG TGG TAG T
/idSp/ GCAGGTCGACTCTAGAGGAT -3'

The DNA template for PCR was a pUC19 vector and following sequence was amplified:
cgggcc tcttcgctat tacgccagct ggcgaaaggg ggatgtgctg caaggcgatt aagttgggta acgccagggt tttcccagtc acgacgttgt aaaacgacgg ccagtgaatt cgagctcggt acccggggat cctctagagt cgacctgc

These are the sequences of the labeled DNA oligonucleotides for co-transcriptional hybridization to the 3'-end of the nascent RNA:

p28_F1_BHQ2: 5'- /5BHQ-2/GTT AAG ATA AGG GAT AGG G/3Cy3Sp/ -3'
p73_F1_Cy3: 5'- GTT AAG ATA AGG GAT AGG G/3Cy3Sp/ -3'
p84_F1_Cy5: 5'- GTT AAG ATA AGG GAT AGG G/3Cy5Sp/ -3'

Minimal 3-way RNA junction construct (85 nt construct) used to monitor relative H20-H22 RNA helical junction conformations (Supplementary Fig. 5a):
The 85 nt construct was obtained by annealing the following three separate RNA strands, each one functionalized with either Cy3, Cy5 or biotin.

p0099A-Cy3 (22 nts): 5'-Cy3-GGCgcac gcaggcgguu ugCCG -3'
p0099B-biotin (29 nts): 5'-Biotin-CGGcaag cuugagucuc guagaggggg gu -3'
p0099C-Cy5 (34 nts): 5'-Cy5-gcccccu ggacgaagac ugacgcucag gugcGCC-3'

Supplementary Figures

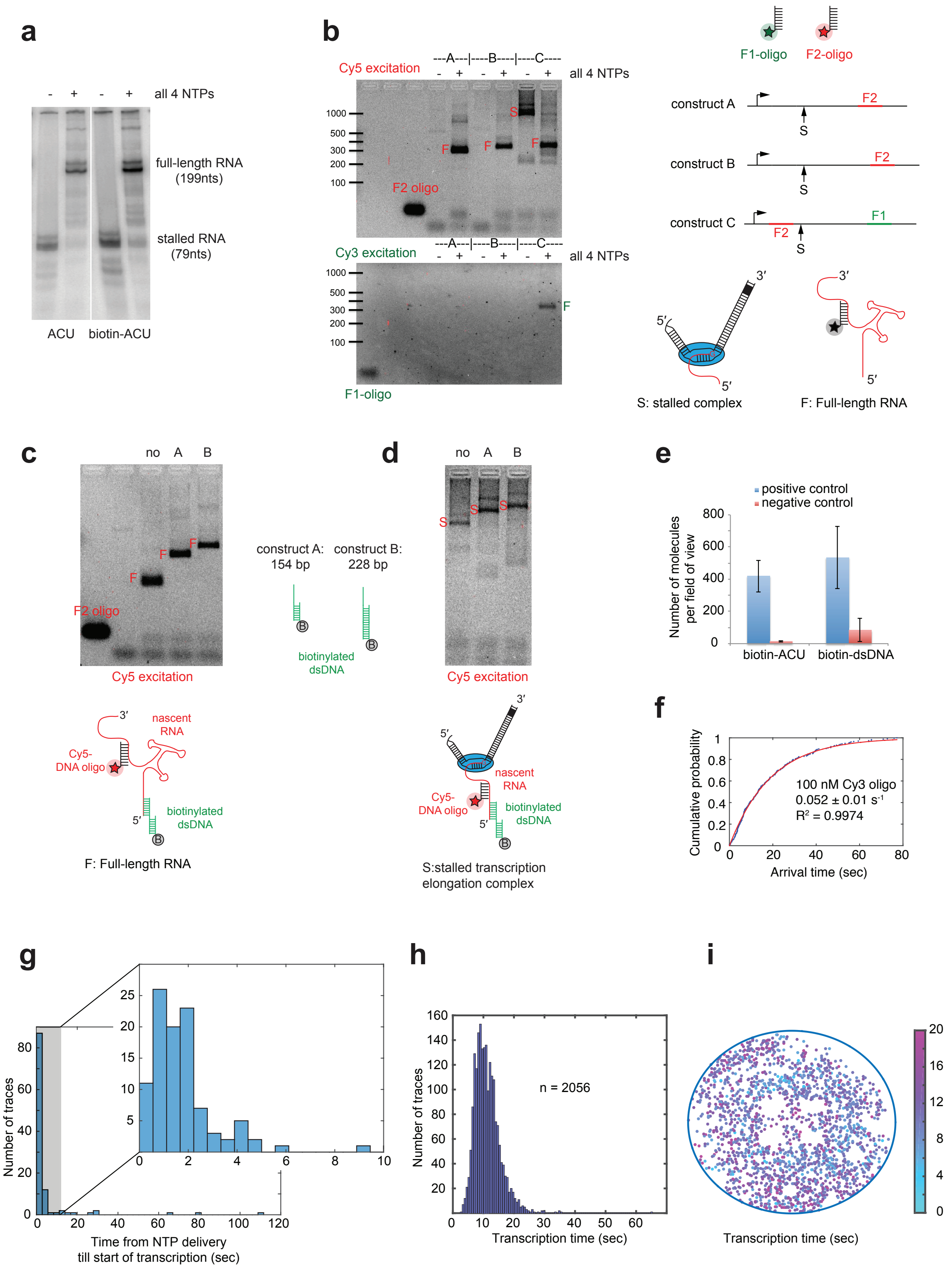

Supplementary Figure 1 | Proof of principle with bulk and single-molecule experiments. a, Stalled transcription complexes can be chased to full-length RNA as illustrated using a 8 % denaturing PAGE gel. The stalled transcription complex was

initiated by mixing a DNA transcription template, *E. coli* RNA polymerase, 10 μM of each ATP, CTP, UTP (+ traces of ^{32}P α-ATP) and either 100 μM ACU or biotin-ACU. To start transcription elongation (chase), rifampicin (to prevent transcription reinitiation) and 1 mM of all the 4 NTPs were added simultaneously. **b,** Stalled transcription elongation complexes can be visualized on a native 4 % agarose gel. Fluorescently labeled DNA oligos can efficiently and specifically hybridize to the stalled RNA (bound to RNAP and DNA template) and/or the full-length RNA depending on the construct design. The nascent RNA efficiently dissociates from the DNA template and the RNA polymerase. **c,d,** A biotinylated double-stranded DNA oligonucleotide containing a single-stranded overhang (biotin-dsDNA) can efficiently hybridize to the full-length RNA (**c**) or the nascent RNA in the stalled transcription elongation complex (**d**). The full-length RNA and the nascent RNA in the stalled transcription elongation complex were visualized on a 4 % agarose gel by hybridizing a short Cy5 DNA oligonucleotide to the 3'-end or the 5'-end of the RNA, respectively. **e,** Immobilization of the biotinylated stalled transcription complex is specific as visualized on a TIRF microscope. The stalled complex was biotinylated either by hybridizing a biotinylated DNA oligo to the 5'-end of the nascent RNA or by priming transcription initiation by a biotin-ACU as described in Methods. In the negative control, the NTPs were omitted during stalled complex formation. **f,** The co-transcriptional binding on-rate of the Cy3 labeled DNA oligo probe binding to the 3'-end of the nascent RNA was determined by fitting a single exponential function to the times between the end of transcription and the binding of the Cy3 DNA oligo, present at a concentration of 100 nM in the ZMW microscope. **g,** Time from NTP delivery till onset of transcription elongation for traces picked in the central part of the SMRT chip. Number of molecules analyzed (*n*) = 111. **h,i,** Demonstration for the large amount of data generated by the ZMW technology during a single experiment. Distribution of transcription times (in seconds) for the 247 nt construct at 20 °C (**h**) and the location of all the analyzed RNA molecules on the SMRT cell, with their transcription times shown as color code (**i**). The time to transcribe an RNA molecule is independent of the location on the chip. Number of molecules analyzed (*n*) = 2056. Source data are provided as a Source Data file.

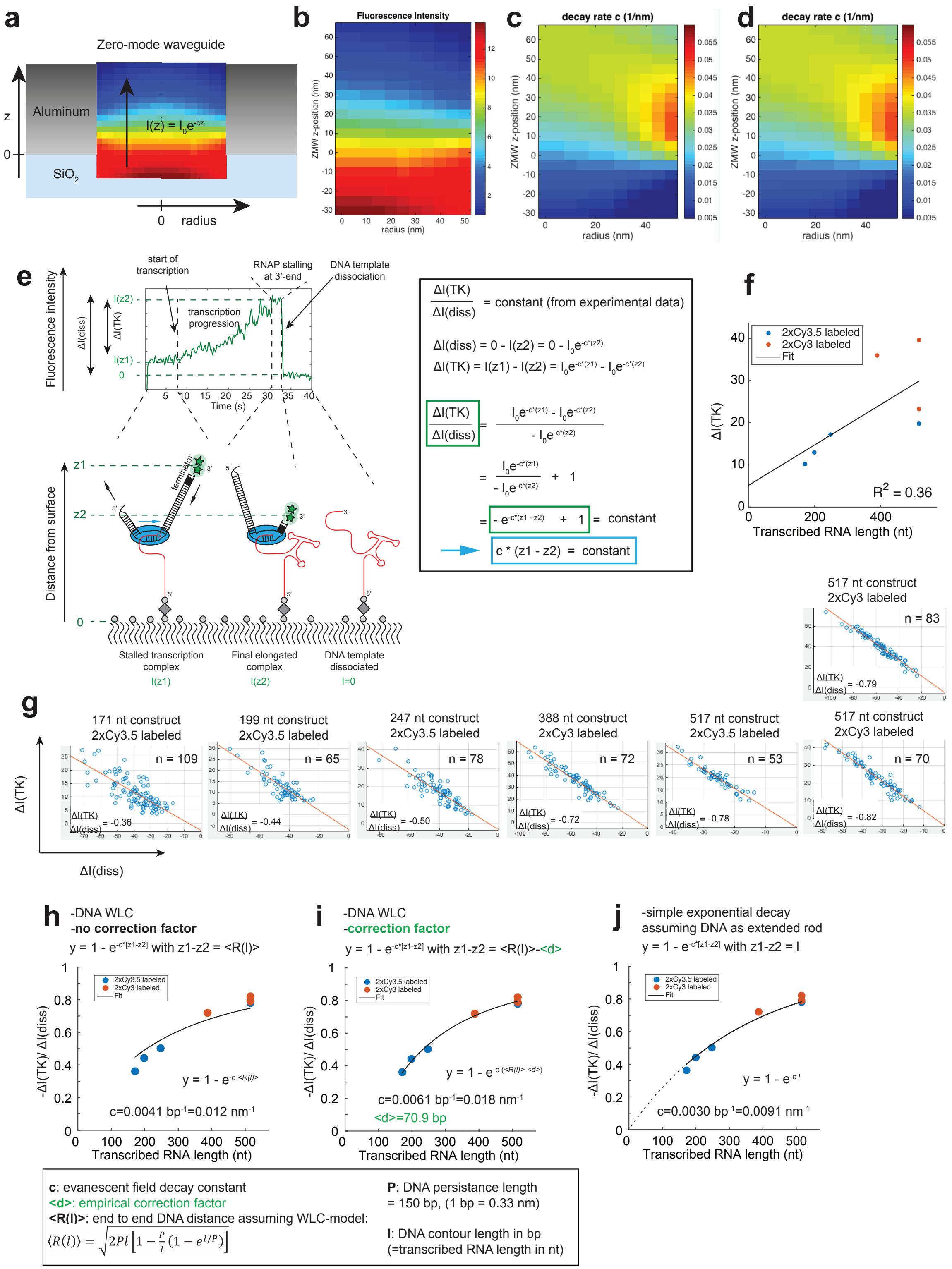

Supplementary Figure 2 | Transcribing within the evanescent field. This figure also supports Supplementary Notes 1 and 2.

a-d, We modeled the excitation field as well as the efficiency of emission using a finite difference time domain approximation to the Maxwell's electromagnetic equations

(https://kb.lumerical.com/en/sp_fluorescence_enhancement.html). Tapered ZMW sidewalls with a bottom and top diameter of 140 nm and 190 nm, respectively and a 90 nm thick Aluminum layer were assumed for modeling. (**a**) Cartoon representation of a typical ZMW well. **b,** Expected fluorescence intensity as a function of the position within the ZMW for emission at 570 nm (Cy3). **c,d,** Calculated fluorescence decay constant as a function of the position within the ZMW for emission at 570 nm (Cy3) (**c**) or 590 nm (Cy3.5) (**d**). Positive and negative *z*-position values correspond to positions within the Aluminum or SiO_2 layers, respectively, with $z = 0$ nm being the Aluminum/ SiO_2 interface; see (**a**).

e-j, Implications of constant normalized fluorescence intensity change during transcription for all the RNA molecules of a specific length: (**e**) Mathematical derivation, why the experimentally observed constant change in normalized fluorescence intensity implies a constant evanescent decay rate constant *c* normal to the surface and a constant average distance travelled by the dyes on the DNA template ($z1$-$z2$) for all the molecules of the same length. **f,** The mean absolute fluorescence intensity change during transcription only poorly correlates with the transcribed RNA length. The mean absolute fluorescence intensity change was determined by fitting the distribution of absolute fluorescence intensity changes of all the RNA molecules of a specific length to a Gaussian distribution; as an example, the 2xCy3-labeled 517 nt construct is shown in Fig. 2f. **g,** Overview of all linear fits of intensity change during transcription versus intensity change upon DNA template dissociation for the different construct lengths (the panel on the top right corresponds to Fig. 2g). The slopes, $\Delta I(TK)/\Delta I(diss)$, are correlated with the construct length and plotted in (**h-j**). **h-j,** The normalized fluorescence intensity change during transcription correlates well with the transcribed RNA length. Fitting the normalized fluorescence intensity change versus transcribed RNA length by a model treating the DNA template by a worm-like chain (WLC) model and without (**g**) or with an empirical transcribed-RNA-length independent correction factor $\langle d \rangle$ (**h**) accounting for the long immobilization tether, the nascent RNA expansion during transcription and other effects, which cannot be described by a simple WLC model accounting only for the DNA template movement. (**j**) A simpler empirical exponential model, which does not describe the DNA as a WLC but describes it as an extended rod, fits our data similarly well and also better extrapolates the relative intensity changes to RNAs of zero length. Number of molecules analyzed (n) = 109, 65, 78, 72, 53, 70, 83 for the data points at 171, 199, 247, 388, 517, 517 and 517 nt transcribed RNA lengths (**f-j**). Source data are provided as a Source Data file.

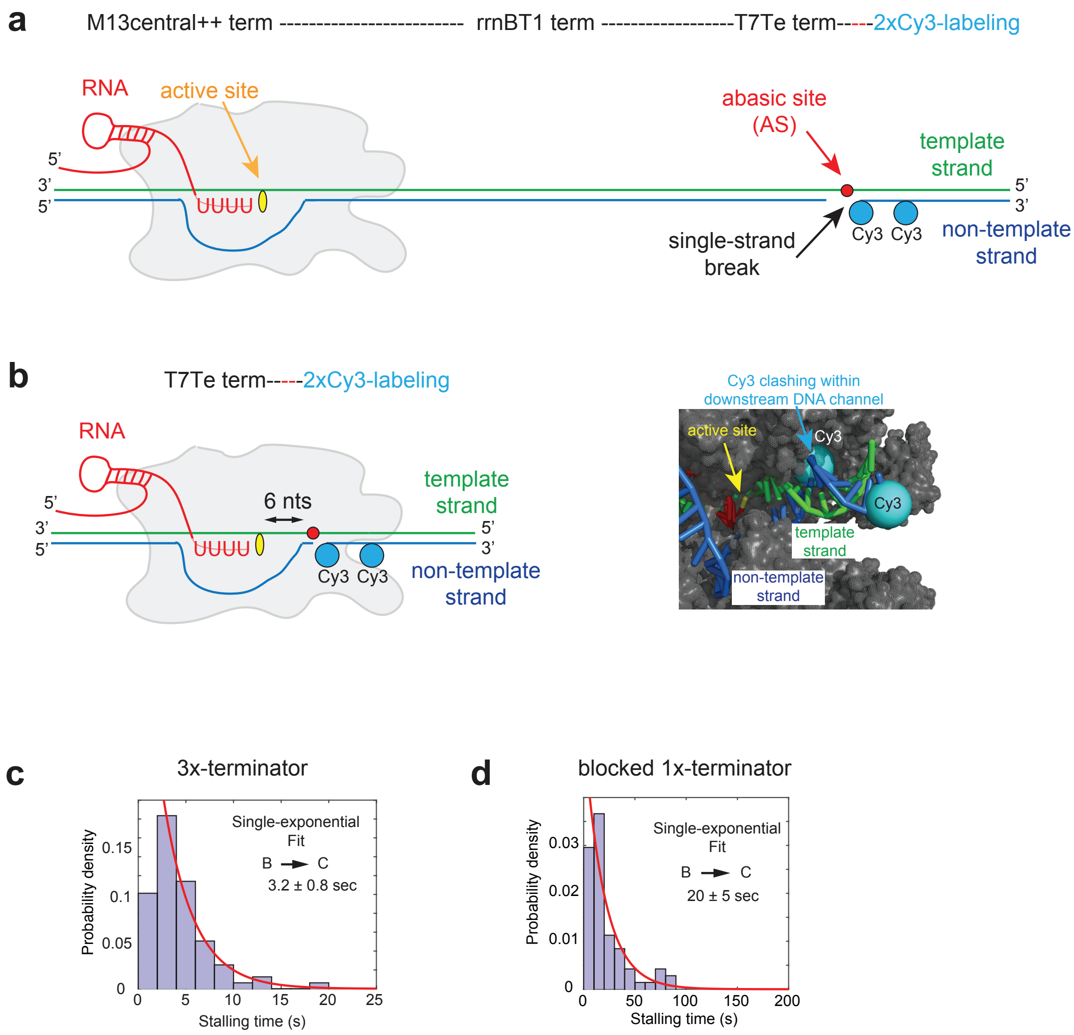

Supplementary Figure 3 | RNA polymerase stalling. a,b, Stalling of RNAP at the 3'-end of two different constructs. In the "3x terminator" construct (**a**), a first *M13central++* terminator is followed by additional *rrnB-T1* and *T7Te* terminators preceding the labeling site. In contrast, the "blocked terminator" construct (**b**) has a single *T7Te* terminator directly followed by the labeling site. The DNA template is labeled by hybridizing a 2xCy3 labeled DNA oligonucleotide to a single-stranded overhang at the 3'-end of the DNA template. In the blocked terminator construct, when the terminating nucleotide is in the active site of the polymerase, the Cy3 dyes are clashing within the downstream DNA channel making termination less efficient. **c,d,** Different representation to Fig. 4c: The residence times at the 3'-end for the 3x-terminator (**c**) and the blocked 1x-terminator (**d**) are plotted separately in a linear scale. Number of molecules analyzed (n) = 79 and 71 for 3x-terminator and blocked 1x-terminator, respectively. Source data are provided as a Source Data file.

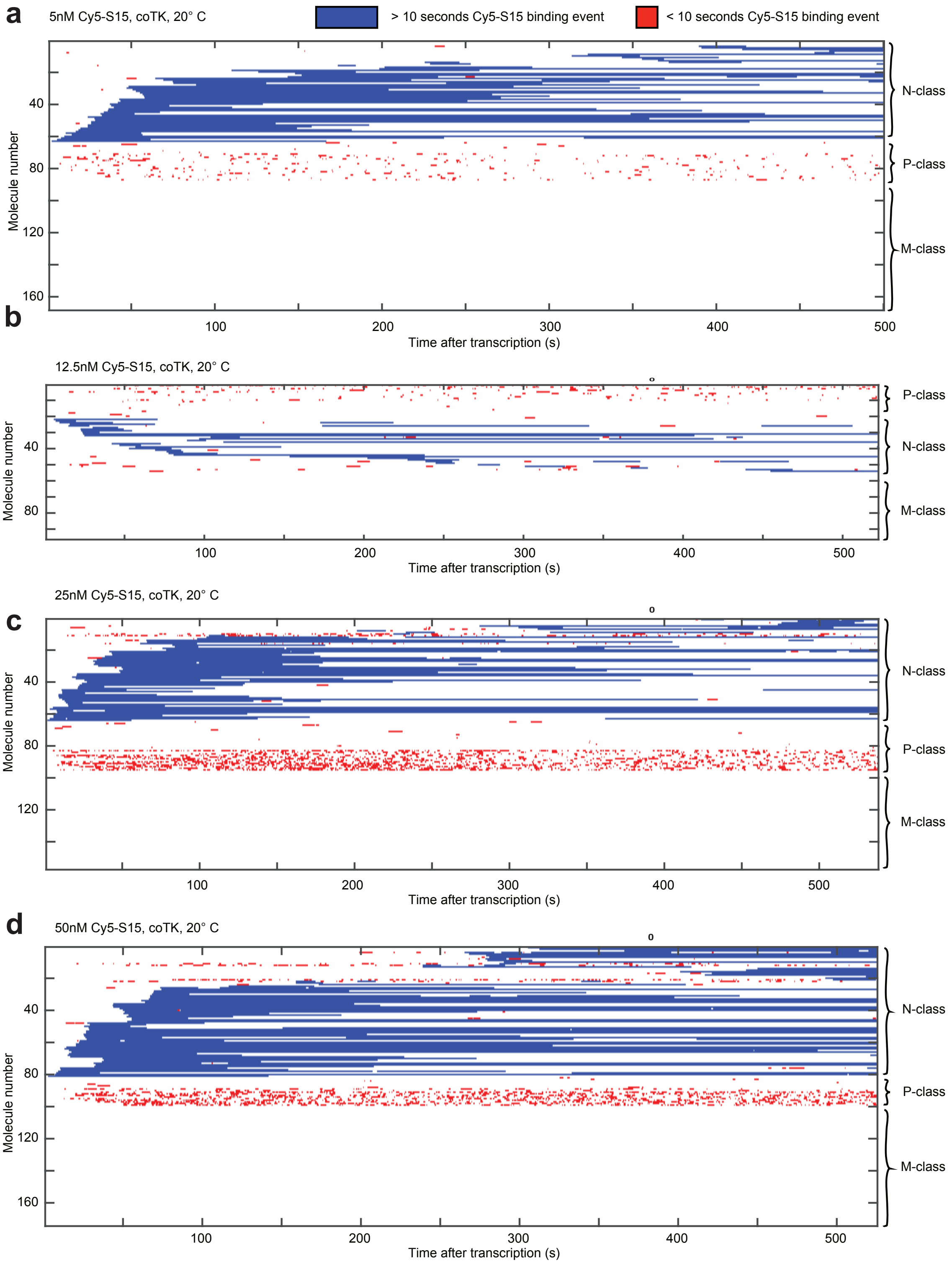
a
5nM Cy5-S15, coTK, 20° C
> 10 seconds Cy5-S15 binding event
< 10 seconds Cy5-S15 binding event
Molecule number
40
80
120
160
Time after transcription (s)
100
200
300
400
500
N-class
P-class
M-class
b
12.5nM Cy5-S15, coTK, 20° C
Molecule number
40
80
Time after transcription (s)
P-class
N-class
M-class
c
25nM Cy5-S15, coTK, 20° C
Molecule number
40
80
120
Time after transcription (s)
N-class
P-class
M-class
d
50nM Cy5-S15, coTK, 20° C
Molecule number
40
80
120
160
Time after transcription (s)
N-class
P-class
M-class

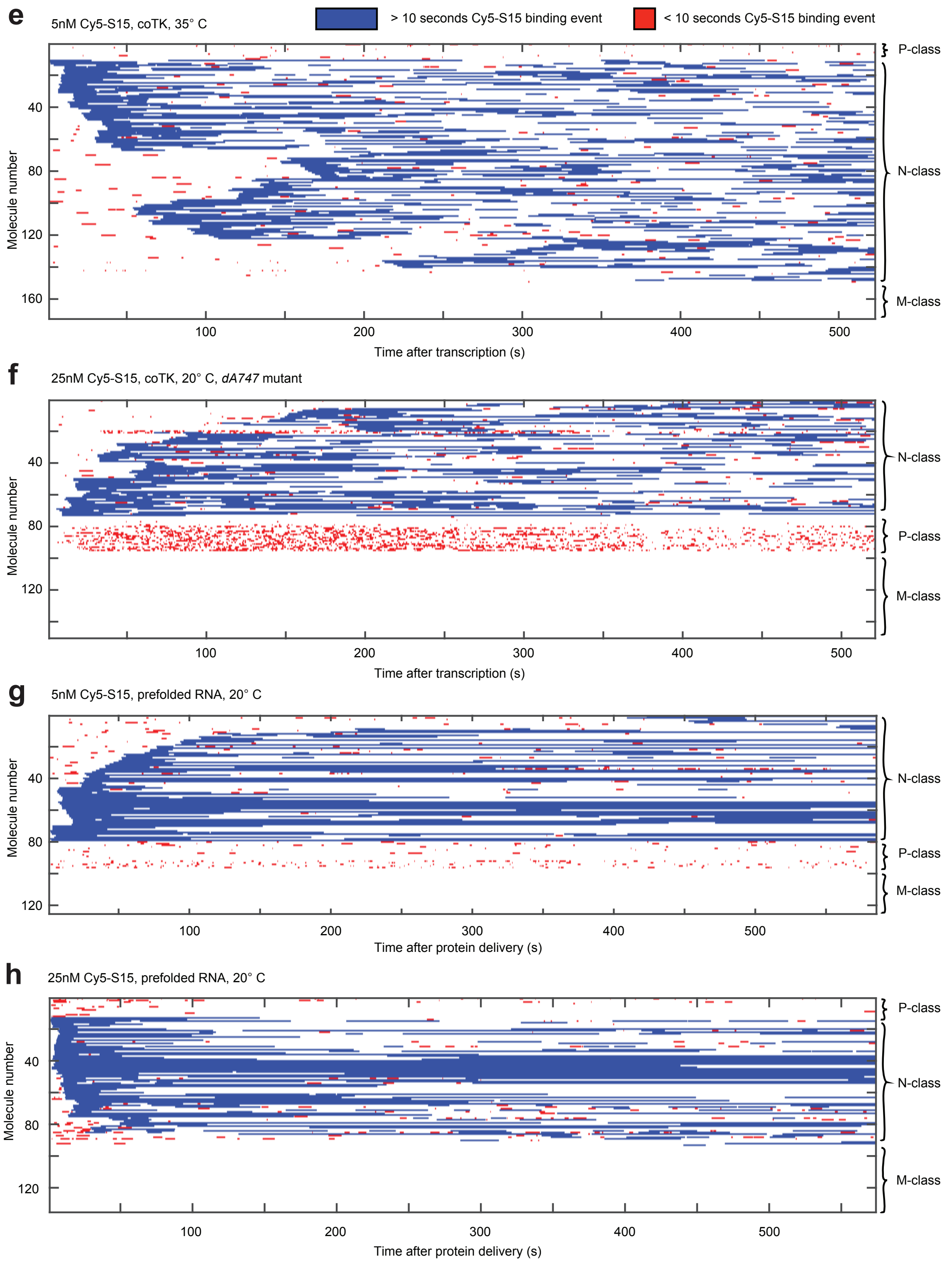

Supplementary Figure 4 | Overview of all analyzed single-molecule traces. a-f, Single-molecule traces are clustered into 3 co-transcriptional RNA folding classes and are post-synchronized to have t = 0 seconds at the end of transcription. **g,h,** The single-molecule traces are clustered into 3 RNA folding classes for a pre-folded RNA

immobilized to the ZMW holes. The Cy5-S15 protein is delivered at t = 0 seconds. Every row represents a single nascent or pre-folded RNA molecule. The single-molecule experiment was performed at the indicated temperature. Number of molecules analyzed (*n*) = 168 (**a**), 96 (**b**), 157 (**c**), 174 (**d**), 172 (**e**), 150 (**f**), 125 (**g**) and 135 (**h**).

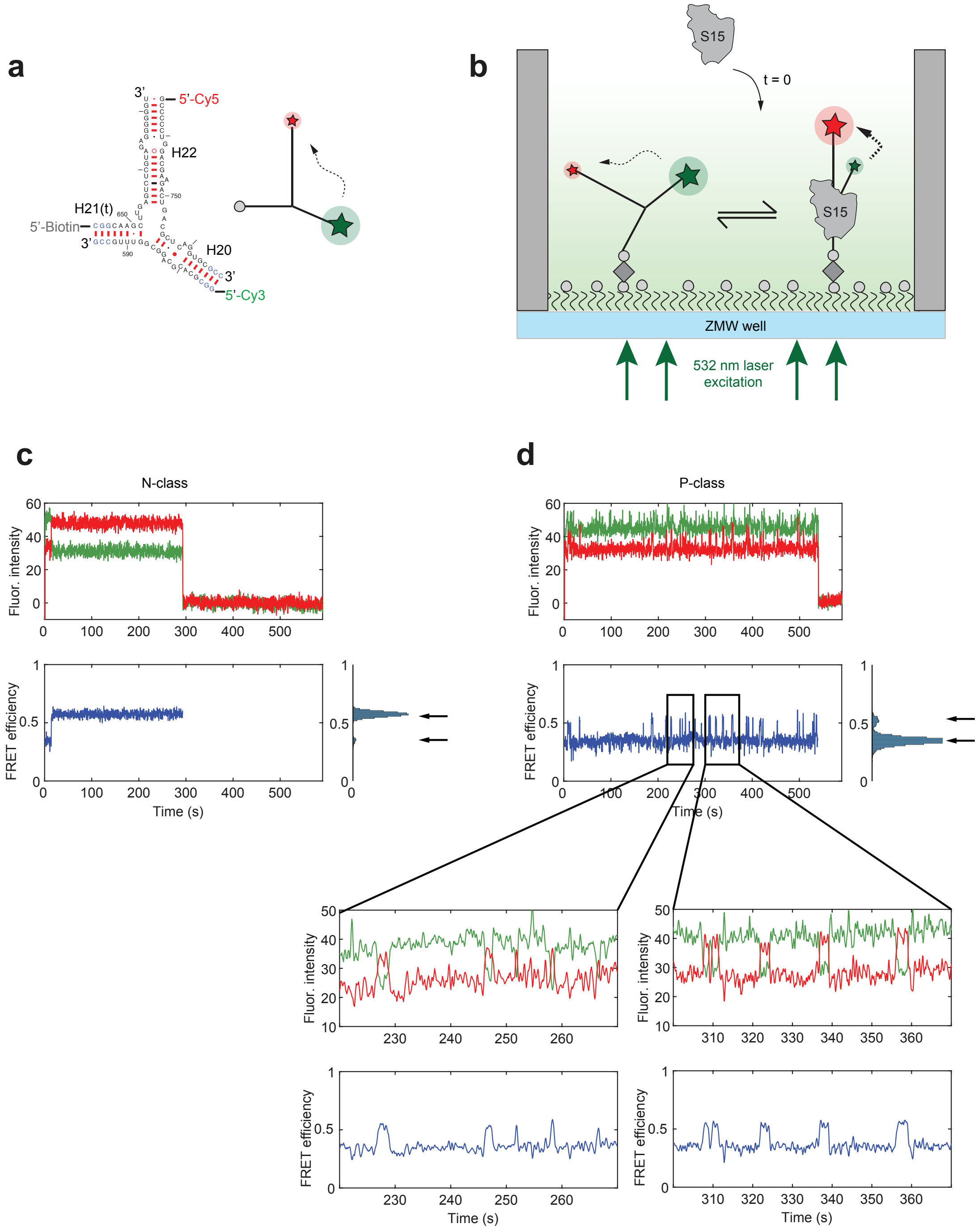

Supplementary Figure 5 | Real-time monitoring of RNA helical junction conformations. Real-time monitoring of the relative helix H20-H22 RNA helical junction conformations shows that the RNA transitions into the native docked conformation both during stable (**c**) and transient (**d**) S15 binding. **a**, Secondary structure of minimal 85 nt 3-way junction central domain construct used. The construct was obtained by annealing three separate RNA strands, each one functionalized with either

Cy3, Cy5 or biotin. **b**, Experimental setup: At t = 0 seconds, 25 nM unlabeled S15 protein was added to the immobilized RNA. **c,d,** Representative single-molecule traces for an N-class (**c**) or P-class (**d**) RNA molecule. The high and low-FRET states represent the docked and undocked conformation of the 3-way junction, respectively and are indicated as arrows on the FRET efficiency histograms. A single laser at 532 nm was used to excite the Cy3 dye.

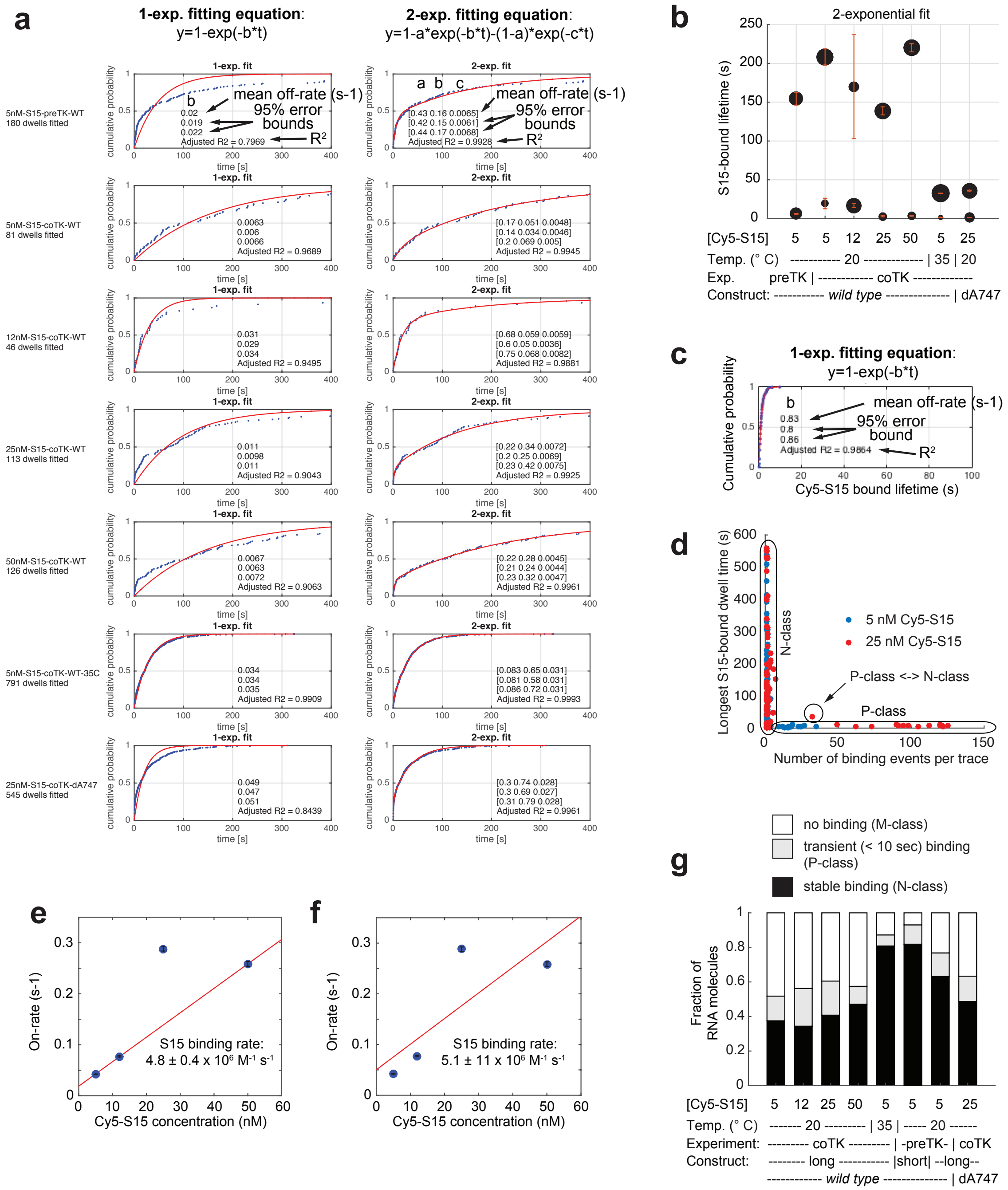

Supplementary Figure 6 | Characterization of S15 binding kinetics. a,b, Average S15-bound lifetimes (**b**), corresponding to the inverse off-rates, of the N-class molecules were obtained by fitting all the S15-bound dwell times to a double exponential function, with the corresponding fits shown in (**a**). In (**b**), the area of the dots is proportional to the population of the two states (corresponding to the pre-exponential factors a and (1-a) of the 2-exponential fit in (**a**), respectively). The error bars in (**b**) represent the 95 % confidence intervals of the fits shown in (**a**). All the S15-bound dwell times of the N-class molecules better fit to a double exponential function than to a single exponential

function, except for the 35° C experiment, which fits equally well to a single exponential function (**a**). The mean off-rates b and c (s-1) and for the 2-exponential fit also the corresponding populations (pre-exponential factors a and (1-a)) are shown with the corresponding 95 % confidence intervals of the fit. Number of molecules analyzed, from top to bottom (**a**) or from left to right (**b**), (*n*) = 69, 60, 28, 61, 76, 138, 70. **c**, Single exponential fit of all the S15-bound dwell-times in the P-class RNA molecules to obtain the average S15-bound lifetime. **d**, Correlating for each trace the longest S15 bound dwell time with the number of S15 binding events reveals the distinct kinetic signature of the RNA molecules corresponding to the N-class or P-class. Number of molecules analyzed (*n*) = 87 and 95 for 5 nM and 25 nM Cy5-S15, respectively. **e,f**, The measured on-rate between the multiple short binding events (s^{-1}) in the P-class RNA molecules is protein concentration dependent. All the arrival times between the short Cy5-S15 binding events were fit to a single exponential function (not shown). The error bars (black; very small) represent the 95 % confidence intervals of those fits. A linear fit (red line) with the equation y=a+b*[Cy5-S15] was used to obtain the S15 second order binding rate constant b (M^{-1} s^{-1}). In **e**, the outlier at 25 nM was omitted for the fit, giving a very similar slope as when fitting including the 25 nM outlier (**f**). **g,** Partitioning of the RNA molecules into 3 folding classes for pre-folded compared to nascently transcribed RNA. This is complementing Fig. 5c. Number of molecules analyzed, from left to right, (*n*) = 168, 96, 157, 174, 172, 65, 125, 150. Source data are provided as a Source Data file.

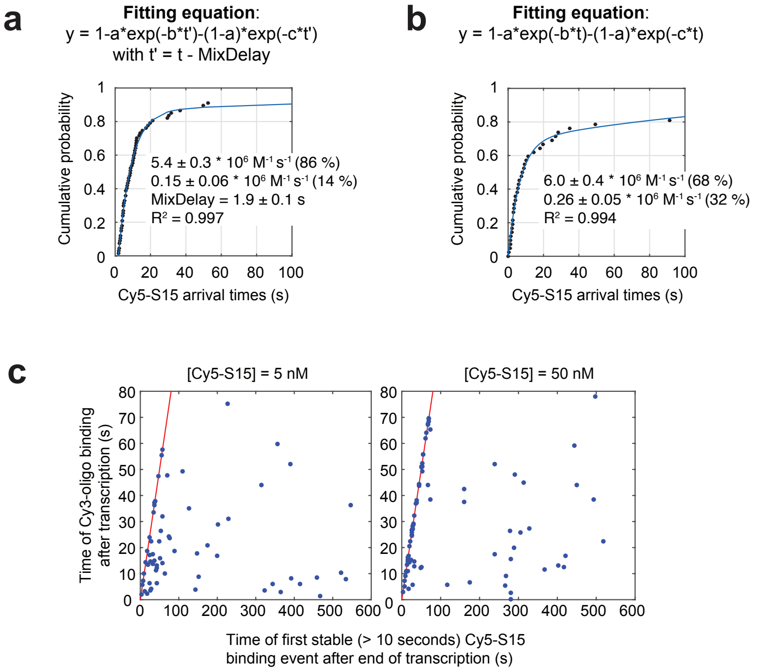

Supplementary Figure 7 | Arrival times for first stable S15 binding event. a,b, On-rate of S15 protein in N-class molecules to pre-folded RNA (**a**) or nascently transcribed RNA (**b**) in which the Cy3-oligo to detect specific Cy5-S15 binding was annealed to the 5'-end of the nascent RNA in order to detect Cy5-S15 protein binding also co-transcriptionally; experimental scheme shown in Fig. 6 was used. The distribution of times from Cy5-S15 protein delivery to first stable binding event (**a**) or times from end of transcription to first stable binding event were fit to a double exponential function. To account for potential mixing delays upon Cy5-S15 protein delivery to the prefolded RNA (**a**), an additional parameter was introduced in the fitting equation. We obtained a mixing delay of 1.9 ± 0.1 seconds. The Cy5-S15 protein concentration was 25 nM in both experiments (**a,b**). Number of molecules analyzed (n) = 67 (**a**) and 42 (**b**). **c,** Detection of the first stable S15 binding event at higher S15 concentrations is often limited by the binding of the Cy3-oligo if hybridized to the 3'-end of the nascent RNA rather than to the 5'-end as used in (**a,b**). The Cy3 oligonucleotide is required for detecting Cy5-S15 binding indirectly by FRET. The plot correlates the time of Cy3-oligo binding with the time of the first stable S15 binding that can be detected (both measured relative from the end of transcription). In many N-class traces, the first stable S15 binding event appears concomitantly with Cy3-oligo binding (appearing on red line). Number of molecules analyzed (n) = 60 and 76 for 5 nM and 50 nM Cy5-S15.

a

b

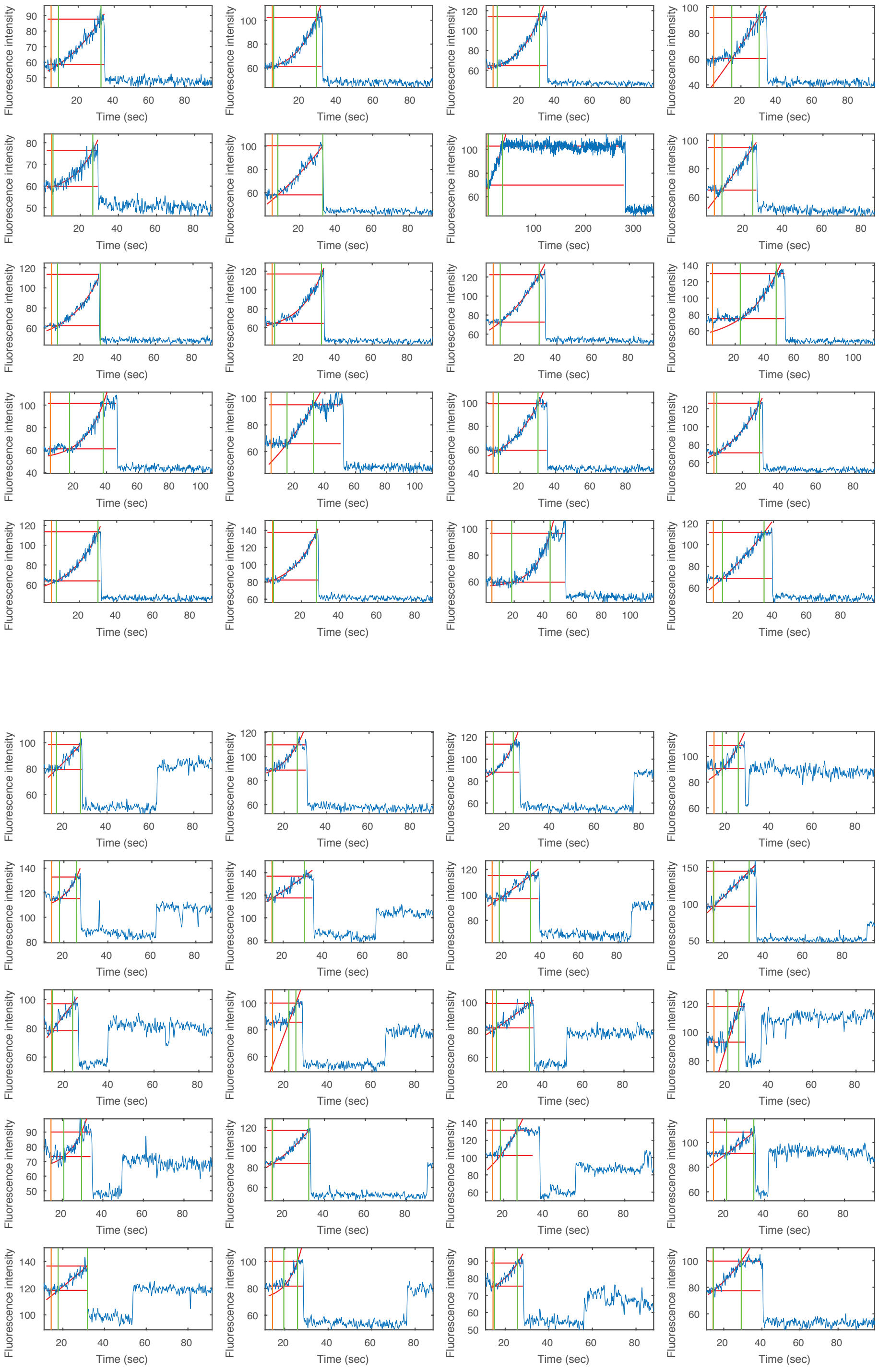

Supplementary Figure 8 | Determination of start and end of transcription. a,b, Representative traces of transcription progression for the 517 nts construct labeled with two Cy3 dyes at the 3’-end (**a**) and the 247 nts construct labeled with two Cy3.5 dyes at the 3’-end (**b**) are shown. Only the Cy3 (**a**) or the Cy3.5 (**b**) channels are shown for clarity. The sudden intensity increase after DNA template dissociation in (**b**) is due to uncorrected bleed-through from a Cy3-labeled DNA oligo binding to the 3’-end of the

nascent RNA (see experimental setup in Fig. 1). In (**a**), no bleed-through is observed into the Cy3 channel from a corresponding Cy5-labeled DNA oligo. To determine start and end of transcription, we fitted horizontal lines to the regions with constant intensity before the start and after the end of transcription. In addition, we fitted the fluorescence intensity increase during transcription elongation to a single exponential function, $y = a * \exp(-b * t) + c$. The fits are shown in red. The intersection points of the two horizonal lines with the exponential fit correspond to the start and end of transcription, respectively and are highlighted as vertical green lines. The time of NTP injection is indicated as a vertical orange line and is at 4.9 seconds (**a**) or 14.4 seconds (**b**).

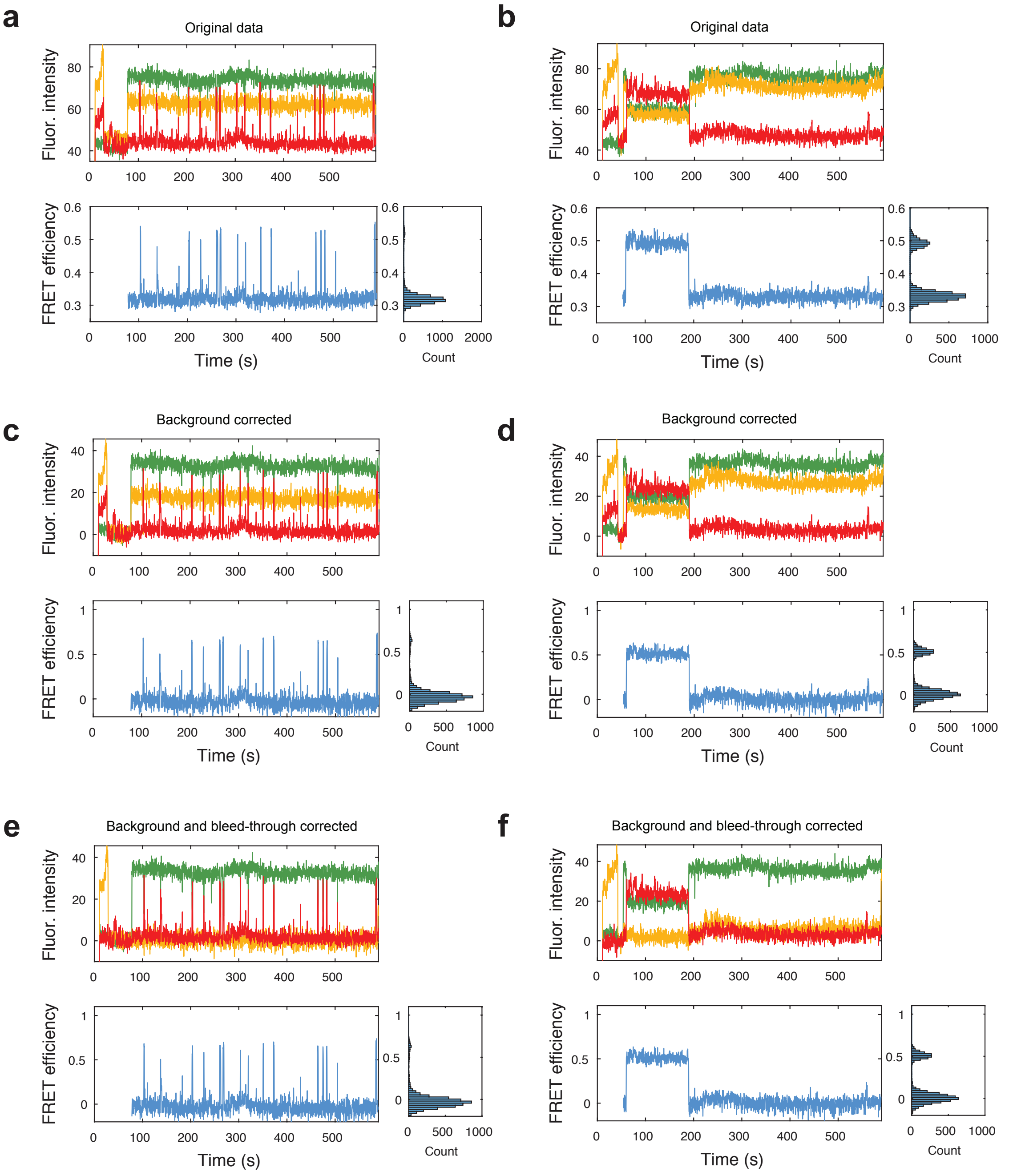

Supplementary Figure 9 | Fluorescence background and bleed-through correction. Fluorescence intensities (top) and calculated FRET efficiencies (bottom) for P-class (**a, c, e**) and N-class (**b, d, f**) RNA molecules. The FRET efficiency is calculated as $E_{FRET} = I_A/(I_A + I_D)$, where I_D and I_A are the apparent fluorescence intensities of the donor and acceptor, respectively. **a,b,** Original traces without background or spectral bleed-through correction. **c,d,** Background corrected but not bleed-through corrected traces. **e,f,** Background and bleed-through corrected traces. The bleed-through was corrected for Cy3->Cy3.5 and Cy3.5->Cy5. A histogram of the FRET efficiency distribution is shown

on the bottom right demonstrating a main bound FRET state of ~0.5-0.6 and a zero-FRET state for the unbound state. The experimental setup and labeling scheme is as described in Fig. 1 with transcription progression (orange), Cy3-oligo (green) and Cy5-S15 protein (red). The reagents were delivered at 14.4 sec.

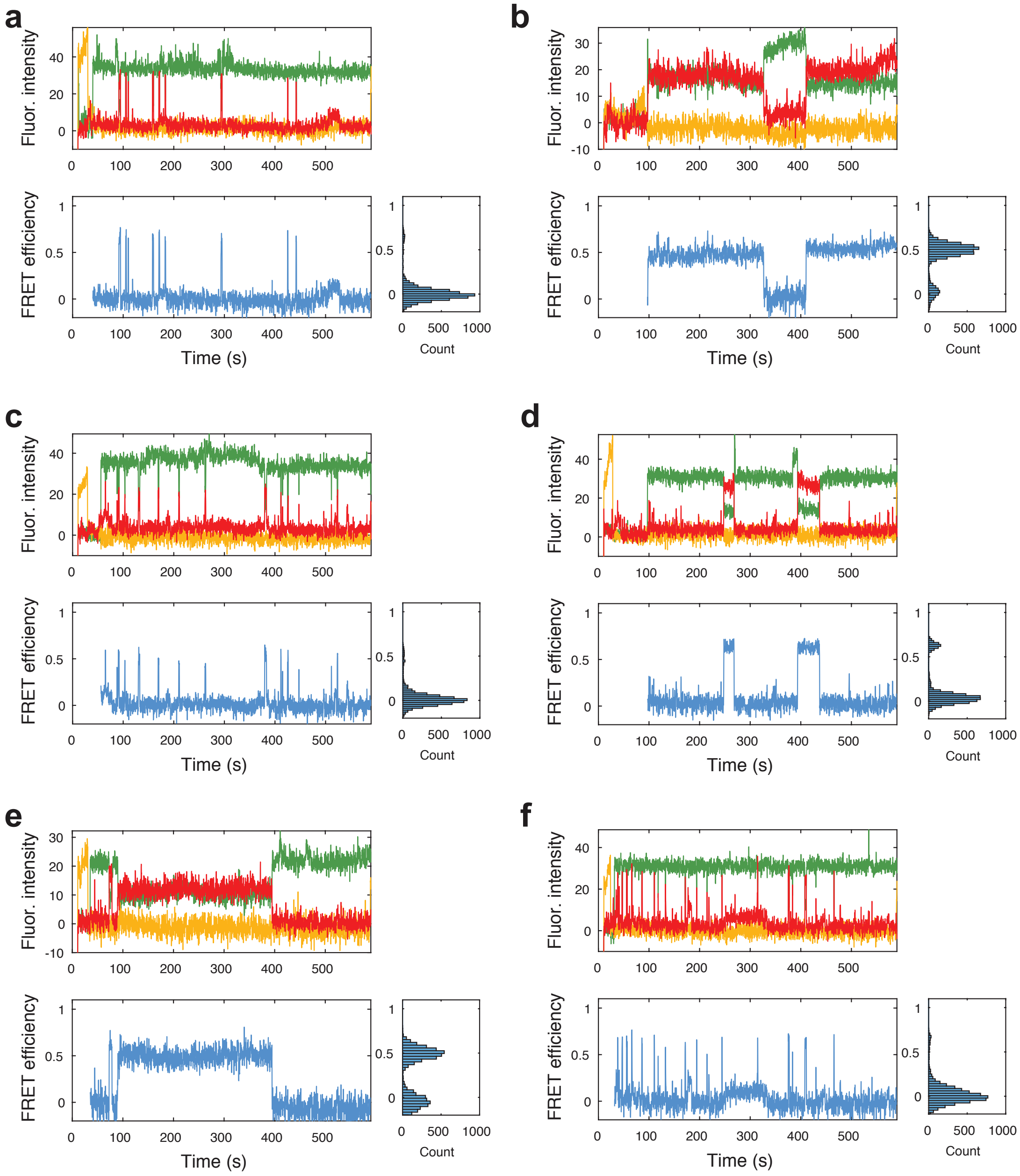

Supplementary Figure 10 | Fluorescence background and bleed-through correction. Representative traces with background and spectral bleed-through correction. Fluorescence intensities (top) and calculated FRET efficiencies (bottom) for P-class (**a, c, f**) and N-class (**b, d, e**) RNA molecules. The FRET efficiency is calculated as $E_{FRET} = I_A/(I_A + I_D)$, where I_D and I_A are the apparent fluorescence intensities of the donor and acceptor, respectively. The FRET efficiency distributions are shown on the bottom right. The experimental setup and labeling scheme is as described in Fig. 1 with transcription progression (orange), Cy3-oligo (green) and Cy5-S15 protein (red). The reagents were delivered at 14.4 sec.

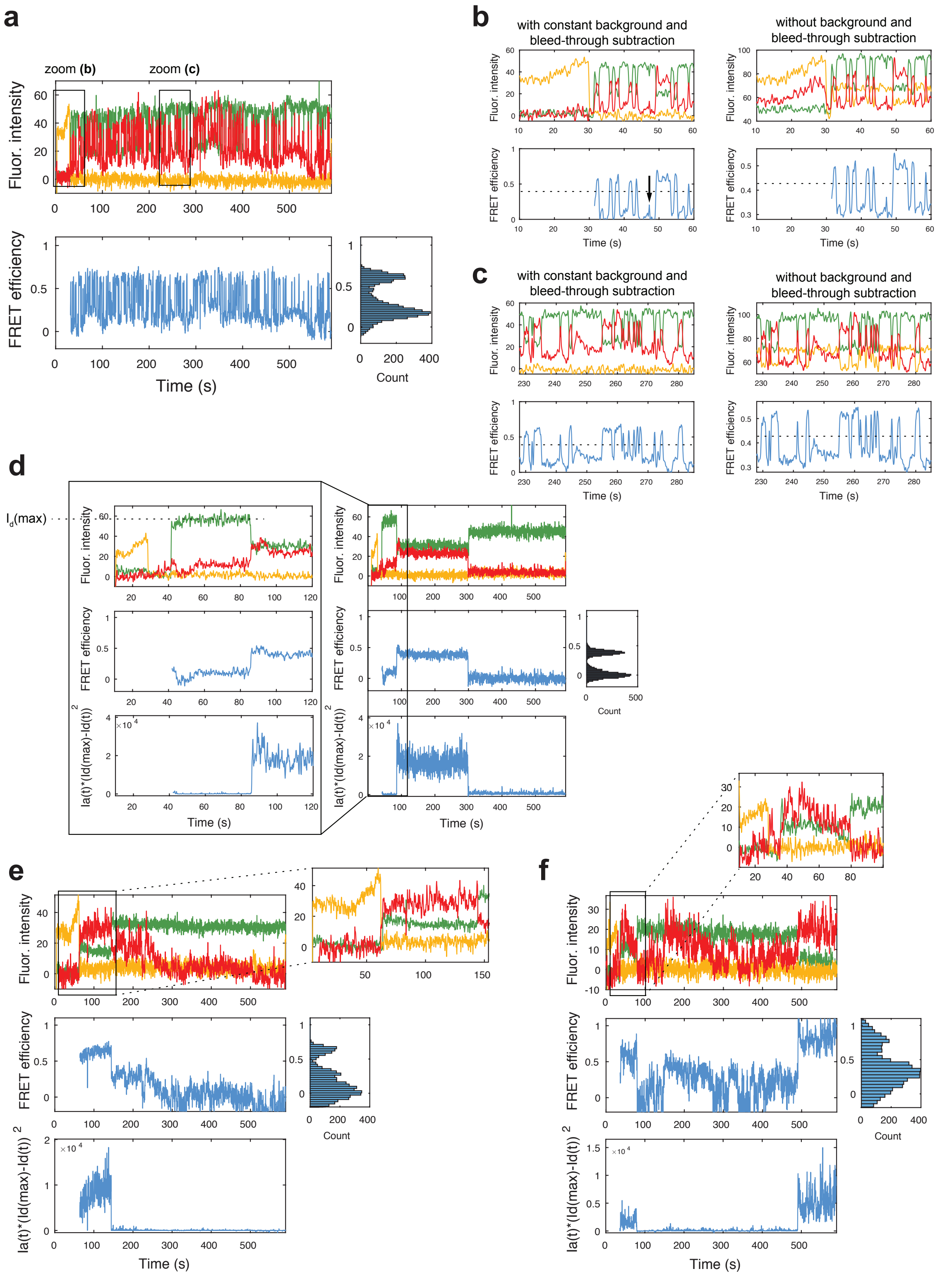

Supplementary Figure 11 | State assignments for traces with significant non-specific Cy5-S15 protein binding. a-c, Representative trace with non-specific Cy5-S15 protein binding **(a)** and zoom into two regions **(b,c)** showing a changing Cy5-intensity

background that is not constant over time which results in a non-flat FRET efficiency baseline also after constant background subtraction (compare zoom-ins in (**b**) and (**c**)). This requires a trace-specific adjustment of the threshold to assign the bound state (dotted line in **b,c**) and subsequent manual inspection to discard wrongly assigned events or recover missed events (shown as arrow), which can be unambiguously assigned due to anticorrelated Cy3 and Cy5 fluorescence intensity changes. In **(b,c)**, the left two panels are with constant background subtraction and spectral bleed-through correction, while the two right panels are without any background and bleed-through correction. We note that the assignment of the S15-protein bound states do not change if not performing background correction. Omitting background correction simply does not allow to use FRET for obtaining quantitative distance information. **d-f**, Assigned states of traces with significant non-specific protein binding can alternatively be unambiguously detected using a different metric than FRET efficiency. The bottom plot in (**d-f**) shows a metric, defined by $I_A(t) * [I_D(t)-I_D(max)]^2$, where $I_D(max)$ is the intensity of the Cy3-oligo in absence of FRET. This allows to unambiguously assign even traces with very strong non-specific protein binding (**f**). The experimental setup and labeling scheme is as described in Fig. 1 with transcription progression (orange), Cy3-oligo (green) and Cy5-S15 protein (red). The Cy5-S15 protein concentrations are 5 nM (**d**), 25 nM (**a,f**) and 50 nM (**e**). The reagents were delivered at 14.0 sec. (**a,f**), 14.4 sec (**d**) and 4.1 sec. (**e**).

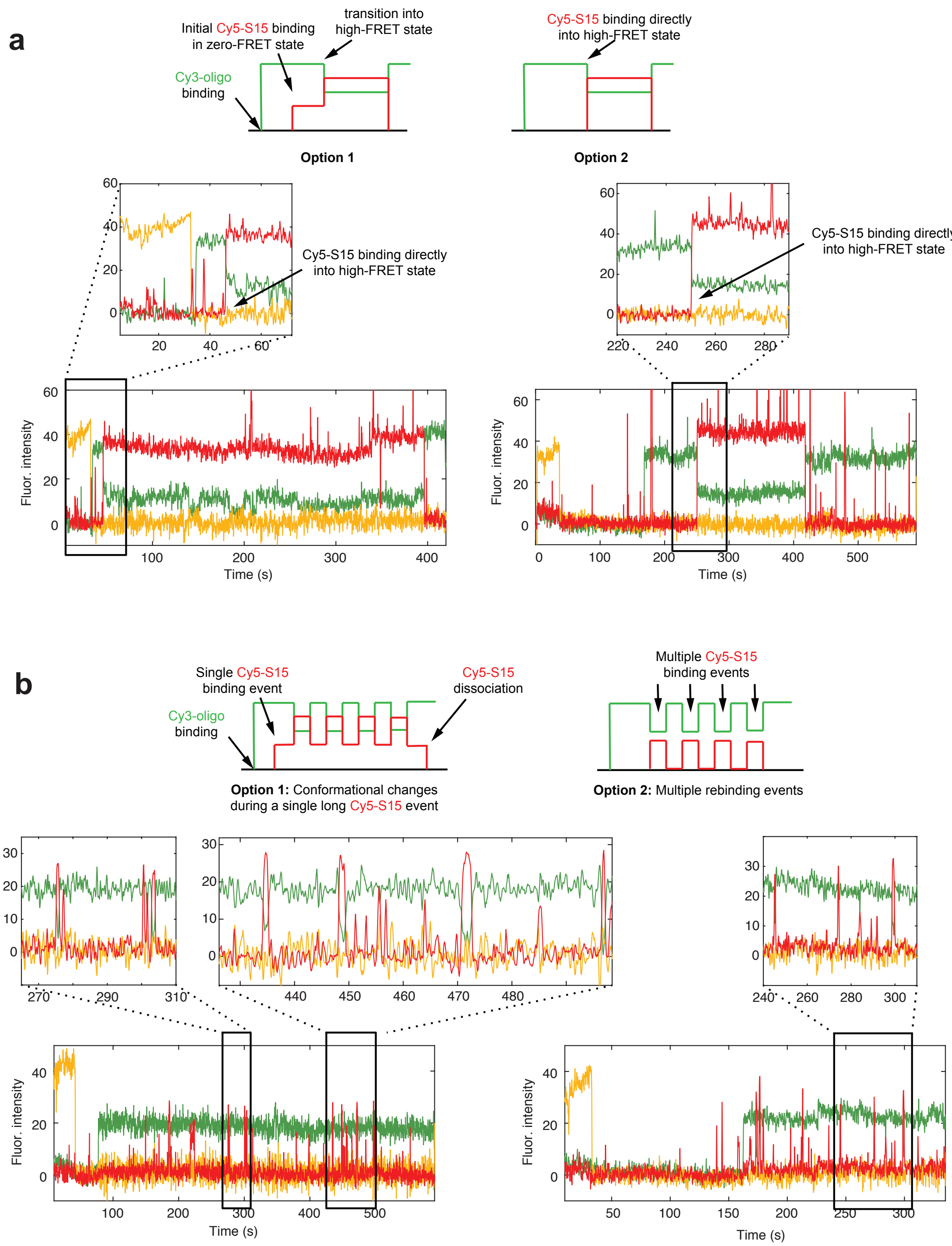

Supplementary Figure 12 | Investigating binding of Cy5-S15 in a zero-FRET state using direct Cy5-S15 protein detection. a,b, Same experimental setup as described in Fig. 1, except that in addition, Cy5-S15 was also excited directly to detect potential Cy5-S15 binding also in a zero-FRET state. Therefore, both 532 nm and 642 nm lasers were used to excite the Cy3 and Cy5 dyes, respectively. To minimize non-specific protein

binding to the surface, we used 2 nM Cy5-S15. (**a**) N-class RNA molecules bind Cy5-S15 directly in a high-FRET state (option 2) and we do not see evidence for initial binding in a low-FRET or zero-FRET state (option 1) as, for example, observed for r-protein S4 binding to the 5'-domain of the 16S rRNA [15]. (**b**) The fast FRET efficiency fluctuations observed for the P-class RNA molecules represent multiple rebinding events (option 2) and not conformational changes during a single long Cy5-S15 binding event (option 1). The reagents were delivered at 12.6 sec.

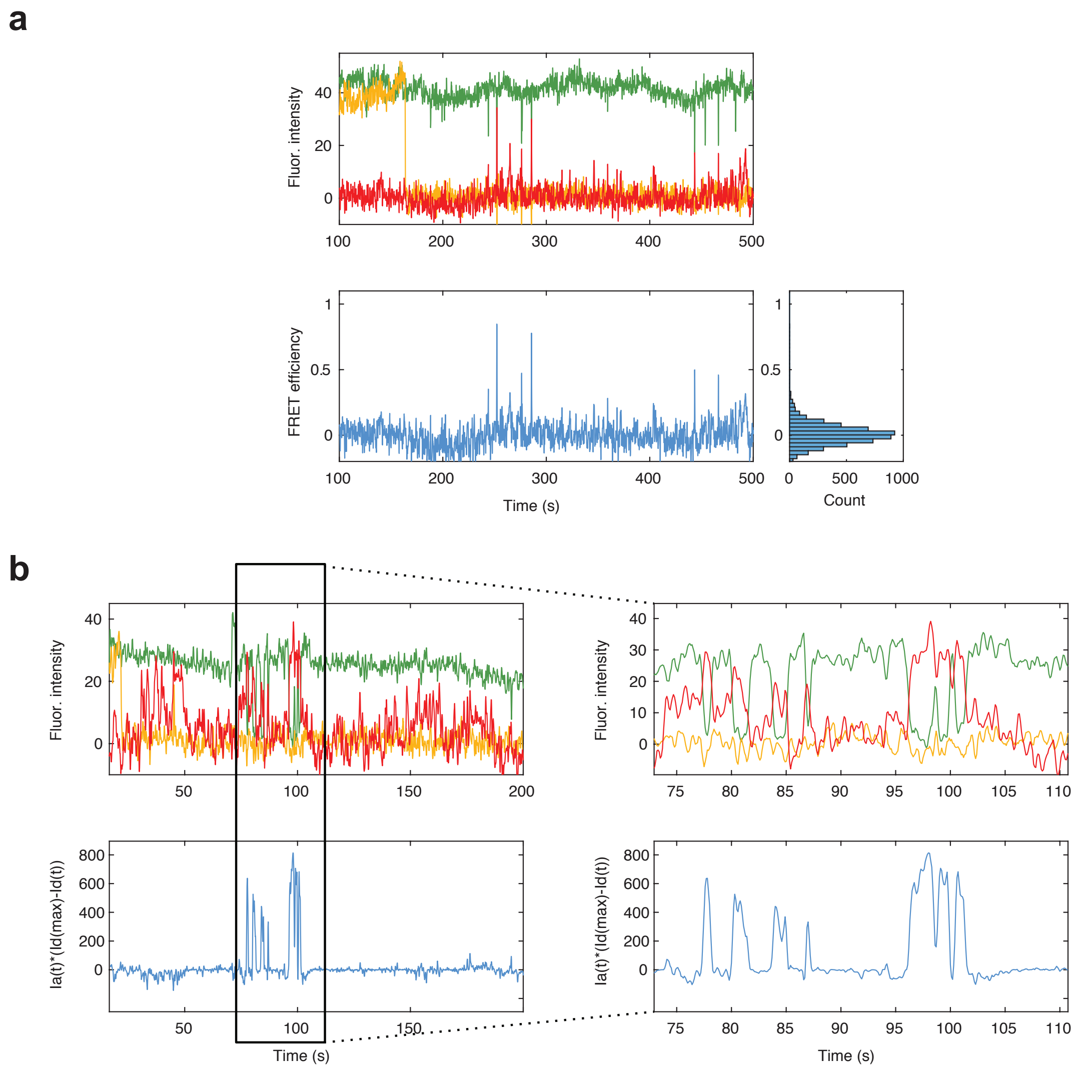

Supplementary Figure 13 | P-class RNA molecules are also found when hybridizing a Cy3-oligo to the 5'-end of the nascent RNA according to the experimental setup shown in Fig. 6a. The top panels show the fluorescence intensities representing transcription (orange), the Cy3-oligo bound to the 5'-end of the nascent RNA (green) and Cy5-S15 protein binding (red). The bottom panels show either the calculated FRET efficiencies (**a**) or $I_A(t)$ * $[I_D(t)\text{-}I_D(max)]$ (**b**), where I_A and I_D are the Cy5 and Cy3 intensities and $I_D(max)$ the intensity of the Cy3-oligo in absence of FRET. This metric is used to assign specific protein binding for traces with significant unspecific Cy5-S15 binding (see Supplementary Fig. 11 for details).

Supplementary References

1 Rubinstein, M. & Colby, R. H. *Polymer physics*. (Oxford University Press, 2016).

2 Russell, R. *et al.* Rapid compaction during RNA folding. *Proc Natl Acad Sci U S A* **99**, 4266-4271, doi:10.1073/pnas.072589599 (2002).

3 Fang, X. *et al.* Mg2+-dependent compaction and folding of yeast tRNAPhe and the catalytic domain of the B. subtilis RNase P RNA determined by small-angle X-ray scattering. *Biochemistry* **39**, 11107-11113 (2000).

4 Zhang, Z., Revyakin, A., Grimm, J. B., Lavis, L. D. & Tjian, R. Single-molecule tracking of the transcription cycle by sub-second RNA detection. *eLife* **3**, e01775, doi:10.7554/eLife.01775 (2014).

5 Floyd, D. L., Harrison, S. C. & van Oijen, A. M. Analysis of kinetic intermediates in single-particle dwell-time distributions. *Biophys J* **99**, 360-366, doi:10.1016/j.bpj.2010.04.049 (2010).

6 Friedman, L. J. & Gelles, J. Mechanism of transcription initiation at an activator-dependent promoter defined by single-molecule observation. *Cell* **148**, 679-689, doi:10.1016/j.cell.2012.01.018 (2012).

7 Senavirathne, G. *et al.* Activation-induced deoxycytidine deaminase (AID) co-transcriptional scanning at single-molecule resolution. *Nat Commun* **6**, 10209, doi:10.1038/ncomms10209 (2015).

8 Mukhopadhyay, J. *et al.* Translocation of sigma(70) with RNA polymerase during transcription: fluorescence resonance energy transfer assay for movement relative to DNA. *Cell* **106**, 453-463 (2001).

9 Batey, R. T. & Williamson, J. R. Interaction of the Bacillus stearothermophilus ribosomal protein S15 with 16 S rRNA: II. Specificity determinants of RNA-protein recognition. *J Mol Biol* **261**, 550-567, doi:10.1006/jmbi.1996.0482 (1996).

10 Serganov, A. *et al.* Role of conserved nucleotides in building the 16 S rRNA binding site for ribosomal protein S15. *J Mol Biol* **305**, 785-803, doi:10.1006/jmbi.2000.4354 (2001).

11 Ridgeway, W. K., Seitaridou, E., Phillips, R. & Williamson, J. R. RNA-protein binding kinetics in an automated microfluidic reactor. *Nucleic Acids Res* **37**, e142, doi:10.1093/nar/gkp733 (2009).

12 Batey, R. T. & Williamson, J. R. Effects of polyvalent cations on the folding of an rRNA three-way junction and binding of ribosomal protein S15. *RNA* **4**, 984-997 (1998).

13 Ha, T. *et al.* Ligand-induced conformational changes observed in single RNA molecules. *Proc Natl Acad Sci U S A* **96**, 9077-9082 (1999).

14 Batey, R. T. & Williamson, J. R. Interaction of the Bacillus stearothermophilus ribosomal protein S15 with 16 S rRNA: I. Defining the minimal RNA site. *J Mol Biol* **261**, 536-549, doi:10.1006/jmbi.1996.0481 (1996).

15 Kim, H. *et al.* Protein-guided RNA dynamics during early ribosome assembly. *Nature* **506**, 334-338, doi:10.1038/nature13039 (2014).
