# Supplementary material for: Real-time assembly of ribonucleoprotein complexes on nascent RNA transcripts
Source: Nat Commun. 2018 Nov 30;9:5087. doi: 10.1038/s41467-018-07423-3 (PMC6269517; doi:10.1038/s41467-018-07423-3)
Supplement: Supplementary file 3 — Description of Additional Supplementary Information [file 41467_2018_7423_MOESM3_ESM.pdf]

Description of Additional Supplementary Files

File Name: Supplementary Data 1
Description: Source data and statistical measures underlying figures.
